# The impact of insulin resistance and glycaemic control on insulin-like growth factor-1 in patients with type 2 diabetes: a cross-sectional study

**DOI:** 10.1186/s40842-024-00202-8

**Published:** 2024-11-23

**Authors:** Hasanain MohammedHuthaifa AbdlWhab, Araz Al-Saffar, Osama Abbas Mahdi, Rafid Badri Alameri

**Affiliations:** 1 Republic of Iraq Ministry of Health, Karbala, Iraq; 2 Al-Nahrain College of Medicine, Al-Kadhimia, Iraq; 3 Faculty of Medicine, University of Mustansiriyah, Baghdad, Iraq

## Abstract

**Background:**

Type 2 diabetes mellitus (T2DM) is a multifaceted metabolic disorder. Over the past decade, the potential role of Growth Hormone (GH) and Insulin-like Growth Factor-1 (IGF-1) in the pathogenesis and progression of T2DM has garnered scientific interest. These hormones, while interrelated, exert differential effects on glucose homeostasis; GH elevates blood glucose levels, whereas IGF-1 sustains insulin secretion and augments insulin sensitivity.

**Objective:**

The study aimed to investigate the impact of insulin resistance and glycaemic control on IGF-1 levels and to assess other risk factors influencing IGF-1 in T2DM.

**Methods:**

A cross-sectional study was conducted at the National Diabetes Centre, Baghdad, Iraq, from May 2020 to May 2021. Sixty patients with T2DM were evaluated for fasting plasma glucose (FPG), GH, IGF-1, HbA1c, HOMA-IR, HOMA-B, and anthropometric measures following a comprehensive history and physical examination, focusing on any variables that could influence their metabolic profile. Patients with Type 1 diabetes mellitus, thyroid disease, pituitary disease, chronic kidney disease, hepatic disease, and pregnancy were excluded from the study.

**Results:**

Patients with poorly controlled diabetes (HbA1c > 8) exhibited significantly elevated IGF-1 levels compared to those with HbA1c < 8 (166 vs. 134, *P* = 0.016). The mean IGF-1 was significantly lower in patients with insulin resistance (IR) compared to those without IR (143 vs. 192, *P* = 0.001), with a significant negative correlation with Body Mass Index (BMI) and a significant positive correlation with HbA1c and Quantitative Insulin Sensitivity Index (QUICKI). Elevated IGF-1 levels were observed with increasing age, duration of T2DM, higher HbA1c, higher QUICKI, and lower BMI. No significant difference was found in IGF-1 values with regards to HOMA-B, fasting insulin, and waist-hip ratio.

**Conclusion:**

Patients with poorly controlled T2DM exhibit higher IGF-1 levels, while those with obesity and high insulin resistance demonstrate lower IGF-1 levels. Further prospective studies are warranted to evaluate the potential of using IGF-1 to reduce insulin resistance and improve metabolic and glycaemic measures in individuals with T2DM and obesity or insulin resistance.

## Introduction

Type 2 Diabetes Mellitus, a multifaceted metabolic disorder, currently afflicts over 463 million individuals globally, with projections indicating a surge to approximately 578 million by the year 2030. The prevalence of this metabolic condition has seen a significant escalation, rising from 6.5% in 2006 to 13.9% in 2015. This serious metabolic disorder primarily emanates from a complex interplay of factors including insulin resistance, disrupted hepatic gluconeogenesis, and a progressive decline in B-cell insulin secretion [1].Over the preceding decade, the scientific community has increasingly focused on the potential involvement of Growth Hormone (GH) and Insulin-Like Growth Factor-1 (IGF-1) in the pathogenesis and progression of Type 2 Diabetes Mellitus (T2DM). These hormones, while interrelated, exert divergent effects on glucose homeostasis. Specifically, GH elevates blood glucose levels, whereas IGF-1 sustains insulin secretion and augments insulin sensitivity. This apparent paradox has catalysed further investigation into the intricate factors influencing this complex interplay. The pursuit of understanding these mechanisms continues to be a significant area of research in the field [2].

### 1-Insulin-like growth factor-1 and growth hormone

Insulin-Like Growth Factor-1 (IGF-1) is a peptide hormone composed of 70 amino acids. It is predominantly produced by the liver, which accounts for approximately 75% of circulating IGF-1, and various other tissues in response to growth hormone [3–5]. IGF-1 shares a structural homology of approximately 50% with insulin. This structural similarity facilitates cross-binding between each hormone and their respective receptors, albeit with differing affinities. IGF-1 receptors exhibit a 1000-fold greater affinity for IGF-1 compared to insulin, while insulin receptors demonstrate a 100-fold greater affinity for insulin than IGF-1 [5–8]. The majority of IGF-1 is bound to Insulin-Like Growth Factor Binding Proteins (IGFBP-1 to 6), with only 0.5-1% of the total IGF-1 pool existing in a free, unbound state, which represents the biologically active form [2, 5, 7–9]. Growth Hormone (GH), also referred to as somatotropin, is a polypeptide hormone comprised of 191 amino acids. It is synthesized and secreted by the somatotrophs located in the anterior pituitary gland, primarily in response to the Growth Hormone-Releasing Hormone (GHRH). Traditionally, GH has been recognized as the principal promoter of linear growth. Moreover, it exerts a fundamental influence on metabolic processes. The metabolic and growth effects of GH are predominantly mediated through Insulin-Like Growth Factor-1 (IGF-1) [11].

### 2- IGF-1, GH and metabolism

Beyond its role as a growth factor, Insulin-Like Growth Factor-1 (IGF-1) performs critical metabolic functions. Generally, it exerts a trophic effect. Within skeletal muscle, IGF-1 facilitates the transportation of amino acids, inhibits protein catabolism, and promotes the uptake and oxidation of fatty acids. Notably, IGF-1 reduces the influx of free fatty acids to the liver, thereby enhancing the insulin-mediated suppression of hepatic gluconeogenesis. In terms of carbohydrate metabolism, IGF-1 operates through the modification of Growth Hormone (GH) and insulin actions. In both adipocytes and hepatocytes, GH stimulates the production of the P85 subunit of PI-3 kinase, leading to the downregulation of p110 and the suppression of insulin action [10–12]. As IGF-1 inhibits GH secretion, insulin action is consequently increased. Postprandial hyperinsulinemia results in the suppression of Insulin-Like Growth Factor Binding Protein-1 (IGFBP-1), thereby increasing free IGF-1.Under conditions of caloric restriction, mammals produce less IGF-1, and its synthesis in the liver becomes refractory to GH stimulation [13, 14]. This process serves to inhibit growth and protein synthesis when nutrients are scarce. Serum IGF-1 levels are reduced in certain conditions such as hypothyroidism, hepatic failure, malnutrition, anorexia nervosa, and poorly controlled type 1 diabetes mellitus [15]. Lower levels of IGF-1 are also associated with hypertriglyceridemia, hypertension, central obesity, and elevated C-reactive protein levels [16–18]. This suggests a correlation between IGF-1 and risk factors for cardiovascular disease [19]. Growth Hormone (GH) serves as a potent stimulator of Insulin-Like Growth Factor-1 (IGF-1) and Insulin-Like Growth Factor Binding Protein-3 (IGFBP3) production in the liver [20]. It enhances protein biosynthesis by promoting amino acid uptake and directly accelerating the transcription and translation of mRNA. Furthermore, GH tends to reduce protein catabolism through its direct effect on adipocytes, stimulating lipolysis and providing a more efficient source of fuel, Acetyl Coenzyme A. This protein-sparing effect is a fundamental function by which GH fosters growth and development. In addition, GH modulates carbohydrate metabolism. An increase in GH levels results in decreased carbohydrate utilization and impaired intracellular glucose influx. Consequently, GH-induced insulin resistance can be attributed to a post-receptor defect in insulin action, leading to glucose intolerance and secondary hyperinsulinism [20–22]. Studies on patients with acromegaly, a condition caused by a GH-secreting pituitary adenoma, have shown that GH hypersecretion has a deleterious effect on glucose homeostasis. Conditions such as impaired glucose tolerance, Type 2 Diabetes Mellitus (T2DM), and metabolic syndrome are frequently observed in these patients. This underscores the critical role of GH in metabolic regulation and the potential implications of its dysregulation [23].

### IGFI, GH and Diabetes Mellitus

In both primary forms of Diabetes Mellitus, a dysregulation of the GH-IGF-1-IGFBP axis is observed. In patients with Type 1 Diabetes Mellitus, there exists an absolute deficiency of insulin in the portal vein [3]. This deficiency results in the downregulation of hepatic GH receptors, accompanied by an increase in GH levels, a phenomenon that can be characterized as hepatic GH resistance [24]. Concurrently, levels of IGF-1 and IGFBP are also diminished [25].Contrastingly, research conducted by Mercado et al. has demonstrated that patients with Type 1 Diabetes Mellitus exhibit normal hepatic GH receptors, as evidenced by low levels of GHBP. However, it has been observed that Type 2 diabetics requiring insulin therapy have lower GHBP levels compared to patients managed with oral antidiabetic agents. This underscores the complex interplay of these hormonal and metabolic factors in the pathophysiology of Diabetes Mellitus [26, 27].

### Homeostatic model assessment of insulin resistance (HOMA-IR), diabetes mellitus and beyond

Insulin resistance is a pivotal factor in the pathogenesis of Type 2 Diabetes Mellitus (T2DM). Moreover, insulin resistance instigates oxidative damage, culminating in endothelial dysfunction. This pathway mediates the detrimental effects of hyperglycemia, such as atherosclerotic diseases. Recent evidence suggests that insulin resistance propels the progression from frailty to prefrailty and contributes to cognitive impairment in individuals with prediabetes and overt diabetes [28].

The euglycaemic hyperinsulinemic clamp, colloquially known as the insulin clamp technique, is universally considered as the gold standard for quantifying insulin sensitivity. This technique involves the infusion of insulin to achieve a state of hyperinsulinemia, while simultaneously infusing glucose to maintain euglycemia. The rate of glucose infusion required to maintain euglycemia provides a direct measure of insulin sensitivity. In contrast, both the Homeostatic Model Assessment of Insulin Resistance (HOMA-IR) and the Triglyceride-Glucose (TyG) index are indirect measures of insulin resistance. They are derived from fasting plasma glucose and insulin levels (for HOMA-IR) or fasting triglyceride and glucose levels (for TyG index). While the insulin clamp technique provides the most accurate measure of insulin sensitivity, it is invasive, labour-intensive, and necessitates specialized equipment and trained personnel [29]. Consequently, it is not practical for large-scale epidemiological studies or routine clinical use. Conversely, HOMA-IR and the TyG index are simple, non-invasive, and cost-effective methods that can be employed in large-scale studies and clinical practice. However, they may not be as accurate as the insulin clamp technique. Some studies suggest that the TyG index is better correlated with the hyperglycemic clamp test than HOMA-IR. Moreover, the TyG index has been found to have stronger associations with arterial stiffness in patients with type 2 diabetes than HOMA-IR [30].

The Homeostasis Model Assessment (HOMA) index, encompassing both HOMA-IR for insulin resistance and HOMA-B for beta-cell function, is a robust tool with a wide array of applications extending beyond the domain of diabetes. It serves as a valuable instrument in the prediction and management of a diverse range of metabolic and cardiovascular conditions [31].The HOMA index has been recognized as a significant prognostic marker for impending metabolic disturbances and cardiovascular events. It facilitates the identification of individuals at risk of developing conditions such as Type 2 Diabetes Mellitus (T2DM), Major Adverse Cardiovascular Events (MACE), arterial hypertension, dyslipidemia, Non-Alcoholic Steatohepatitis (NASH), and cancer. This holds true even for ostensibly healthy individuals and those presenting with hallmarks of metabolic syndrome [29]. Furthermore, the HOMA index can be employed to predict the incidence of pre-diabetes subtypes. An increase in HOMA-IR has been associated with an elevated risk of isolated Impaired Fasting Glucose (iIFG), isolated Impaired Glucose Tolerance (iIGT), combined impaired fasting glucose and impaired glucose tolerance (CGI), and Diabetes Mellitus (DM).

Elevated HOMA-IR is identified as an early predictor of new-onset Chronic Kidney Disease (CKD), irrespective of HbA1c levels in non-diabetic individuals. Moreover, the HOMA index can provide insights into insulin sensitivity and beta-cell function in both diabetic and non-diabetic individuals, thereby aiding in the identification of those at risk of developing diabetes. This underscores the broad applicability and utility of the HOMA index in the realm of metabolic health [28, 31–34].

Hyperglycemia, is a significant contributor to various adverse events. It has profound implications in cardiovascular health and hospitalization, as well as in the progression from a state of pre-frailty to frailty [28, 31, 35] A recent study conducted on patients with Ischemia and Nonobstructive Coronary Arteries (INOCA) assessed the impact of the stress hyperglycemia ratio (SHR) on the risk of rehospitalization for chest pain. The SHR is defined as the ratio of mmol/L blood glucose and % HbA1c. The findings indicate that patients with an SHR greater than 1 faced a significantly higher risk of rehospitalization for chest pain at the 1-year follow-up, compared to patients with an SHR of 1 or less [35].

In addition to its cardiovascular implications, hyperglycemia has also been found to play a critical role in the transition from pre-frailty to frailty [28]. This underscores the importance of effective management and monitoring of blood glucose levels to mitigate these risks. Furthermore, the American Diabetes Association (ADA) and the European Association for the Study of Diabetes (EASD) have updated their recommendations on the management of hyperglycemia. These updates emphasize the use of glucagon-like-peptide 1 (GLP-1) receptor agonists or sodium–glucose cotransporter 2 (SGLT2) inhibitors to reduce major adverse cardiovascular events (MACE), hospitalization for heart failure (hHF), cardiovascular death, or chronic kidney disease (CKD) progression.

### Knowledge gap

In patients with type 2 diabetes, several variables could affect IGF-1 levels such as insulin resistance, BMI, glycaemic control, inflammatory cytokines, IGFBPs. However, these parameters have not been examined in depth and there is paucity of studies that investigate them in Iraqi population.

### Aim of study

To examine the effect of Insulin resistance and glycaemic control on IGF-1 and asses other risk factors that affect the IGF-1 levels in type 2 diabetes mellitus.

## Patients and methods

### Study design and data collection time

This was a cross sectional study conducted in the National Diabetes Centre, Baghdad, Iraq, from May 2020 to May 2021.

### Study patients and sample size

Seventy patients with type 2 diabetes were recruited from May 2020 to May 2021. 60 patents were included, and 10 patients were excluded.

**The inclusion criteria**

1. Type 2 diabetes mellitus
2. Age 19–75 years

**Exclusion criteria**

1. Type 1 diabetes mellitus
2. Thyroid disease
3. pituitary disease
4. chronic kidney disease
5. Hepatic disease
6. Pregnancy

Each enrolled patient was investigated for fasting plasma glucose (FPG), GH, lGF-1 and HbA1c, HOMA-IR, HOMA-B after a full history and physical examination focusing on any variable that could affect their metabolic profile.

### Anthropometric and metabolic assessment

During each clinical visit, the participants’ height and weight were recorded while they were dressed in light attire. The Body Mass Index (BMI) was subsequently calculated using the formula: weight (in kilograms) divided by the square of height (in meters). Waist and hip circumferences were measured utilizing a fabric tape measure, and the waist-to-hip ratio was subsequently computed. Serum Fasting Plasma Glucose (FPG) levels were determined using an enzymatic method with hexokinase (Cobas C 111), along with fasting triglyceride levels. Serum Haemoglobin A1c (HbA1c) levels were quantified using High-Performance Liquid Chromatography (HPLC).

### Growth hormone assay

The Electrochemiluminescence Immunoassay (ECLIA) is utilized by Cobas e 411 immunoassay analyzers. This method operates on the Sandwich principle. During the first incubation, a 40 µL sample, a biotinylated monoclonal human Growth Hormone (hGH) specific antibody, and a polyclonal hGH specific antibody labelled with a ruthenium complex form a sandwich complex. In the second incubation, streptavidin-coated microparticles are added, and the complex becomes bound to the solid phase via the interaction of biotin and streptavidin. The reaction mixture is then aspirated into the measuring cell, where the microparticles are magnetically captured onto the surface of the electrode. Subsequently, unbound substances are removed with ProCell/ProCell M. The application of a voltage to the electrode induces chemiluminescent emission, which is measured by a photomultiplier. The serum is collected using standard sampling tubes or tubes containing separating gel. The measuring range for males is 1-2.47 ng/ml, while for females, it is 1-9.88 ng/ml.

### Insulin-like growth factor 1 (IGF-1) assay

The Electrochemiluminescence Immunoassay (ECLIA) is employed by Cobas e 411 immunoassay analyzers, operating based on the Sandwich principle. Serum samples are collected utilizing either standard sampling tubes or tubes containing a separating gel. The measurement range is determined in accordance with the age and sex of the individual and is expressed in nanograms per milliliter (ng/ml).

### Insulin assay

The Electrochemiluminescence Immunoassay (ECLIA), utilized by Cobas e 411 immunoassay analyzers, operates on the Sandwich principle. Serum samples are procured using either standard sampling tubes or tubes that contain a separating gel. The assay has a measurement range of 2.6–24.9 mIU/ml. This range is critical for the accurate quantification and assessment of various biomarkers in the serum samples.

### Homeostasis model assessment (HOMA-IR, HOMA-B) calculations

In this study, the Homeostatic Model Assessment (HOMA) method, developed in 1985 by Matthews and colleagues, was employed. This method is considered the gold standard for evaluating insulin resistance via the hyperinsulinemic-euglycemic clamp technique. A noteworthy correlation has been documented between the insulin resistance index as calculated by HOMA and the hyperinsulinemic-euglycemic clamp [36]. However, it is important to note that currently, there is no established cut-off value for HOMA in Iraq.

$$\begin{gathered}\:HOMA - IR = Fasting\:insulin\:(mIU/ml)\: \hfill \\\,\,\,\,\,\,\,\,\,\,\,\,*\:Fasting\:glucose\:(mg/dl)/405 \hfill \\ \end{gathered}$$

$$\:HOMA-B=\:(360\:*\:Fasting\:insulin/Fasting\:glucose-63)$$

### Quantitative insulin sensitivity check index (QUICKI) calculation

This is identical to the simple equation form of the HOMA model in all comparative respects, except that QUICKI uses a log transform of the insulin glucose product.

$$\:QUICKI\hspace{0.17em}=\hspace{0.17em}1\:/log\:\left(Fasting\:glucose\right)\hspace{0.17em}+\hspace{0.17em}log\:\left(Fasting\:insulin\right)$$

$$\:QUICKI\:index\hspace{0.17em}>\hspace{0.17em}0.45\:is\:probably\:healthy$$

$$\:QUICKI\:index\:0.3\--0.45\:might\:be\:insulin\:resistance.$$

$$\:QUICKI\:index\hspace{0.17em}<\hspace{0.17em}0.3\:might\:be\:diabetic.$$

### Ethical considerations and official approvals

Verbal consent was obtained from each patient before collecting data, and the information was anonymous. Each name is given an identification code.

**Administrative approvals were granted from the following**

1. The Council of Iraqi Board of Medical Specialization.
2. Approval and agreement of the national diabetes centre, Baghdad.

### Statistical analysis

All computational statistical analyses were conducted utilizing Microsoft Excel 2010 and the Statistical Package for the Social Sciences (SPSS) software, version 23. The outcomes were articulated as the mean and standard deviation (SD) for continuous variables that followed a normal distribution. For categorical variables, comparisons were made using the independent t-test. The relationships between various continuous variables were evaluated using Spearman’s correlation analysis and linear regression. A p-value of ≤ 0.05 was deemed statistically significant, while a p-value at 0.01 was considered to denote high statistical significance.

## Results

**Fig. 1:**
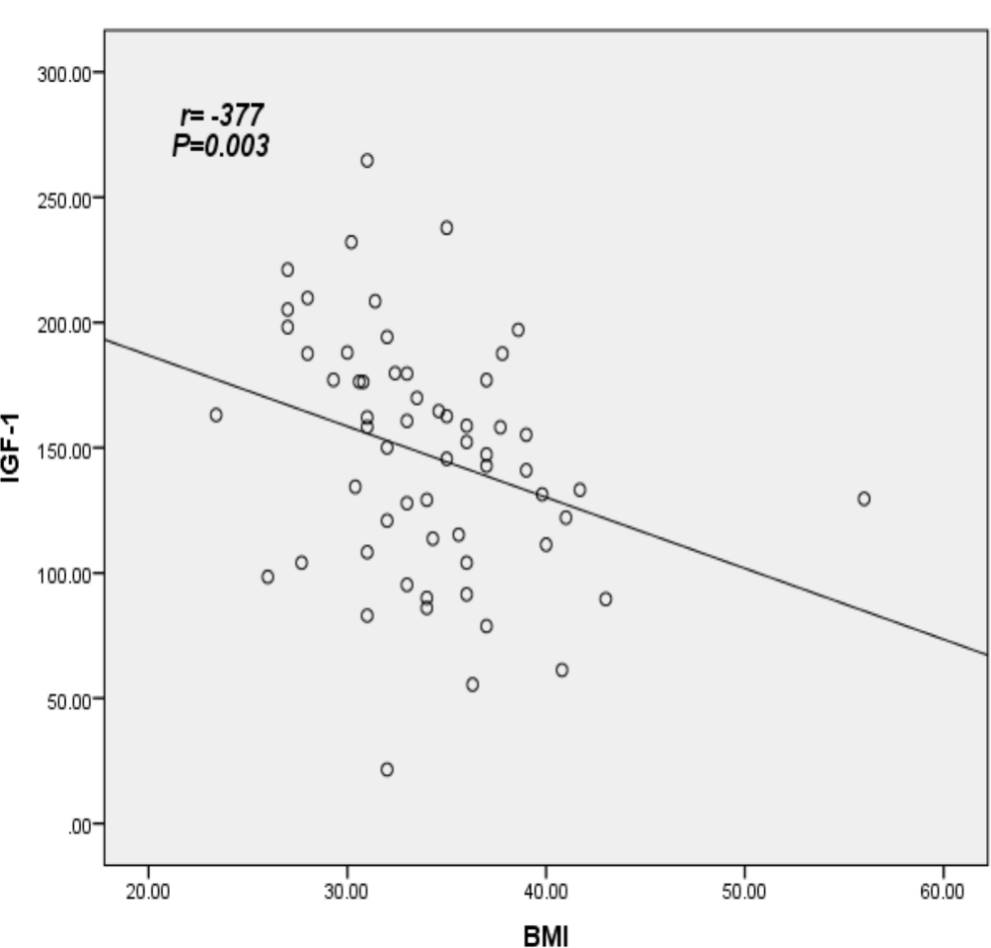
Correlation between IGF-1 and factor related diabetes BMI, using Spearman’s rank correlation coefficient; *P significant at level ≤ 0.05

**Fig. 2:**
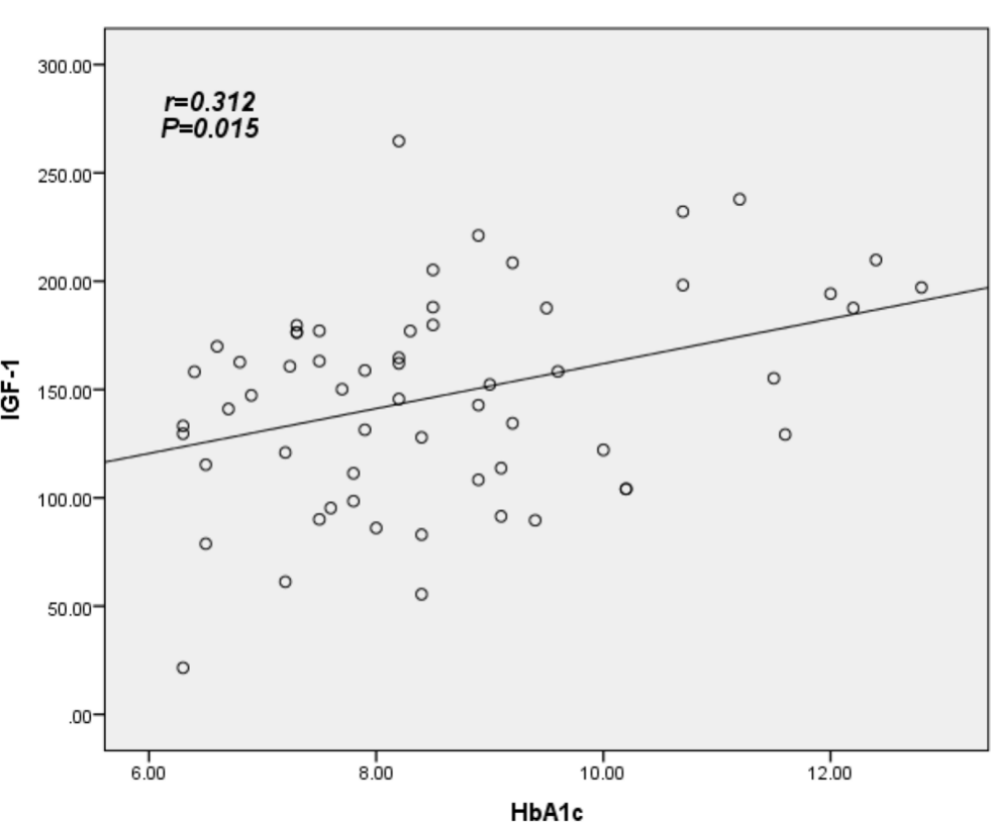
Correlation between IGF-1 and factor related diabetes HbA1c, using Spearman’s rank correlation coefficient; *P significant at level ≤ 0.05

**Fig. 3:**
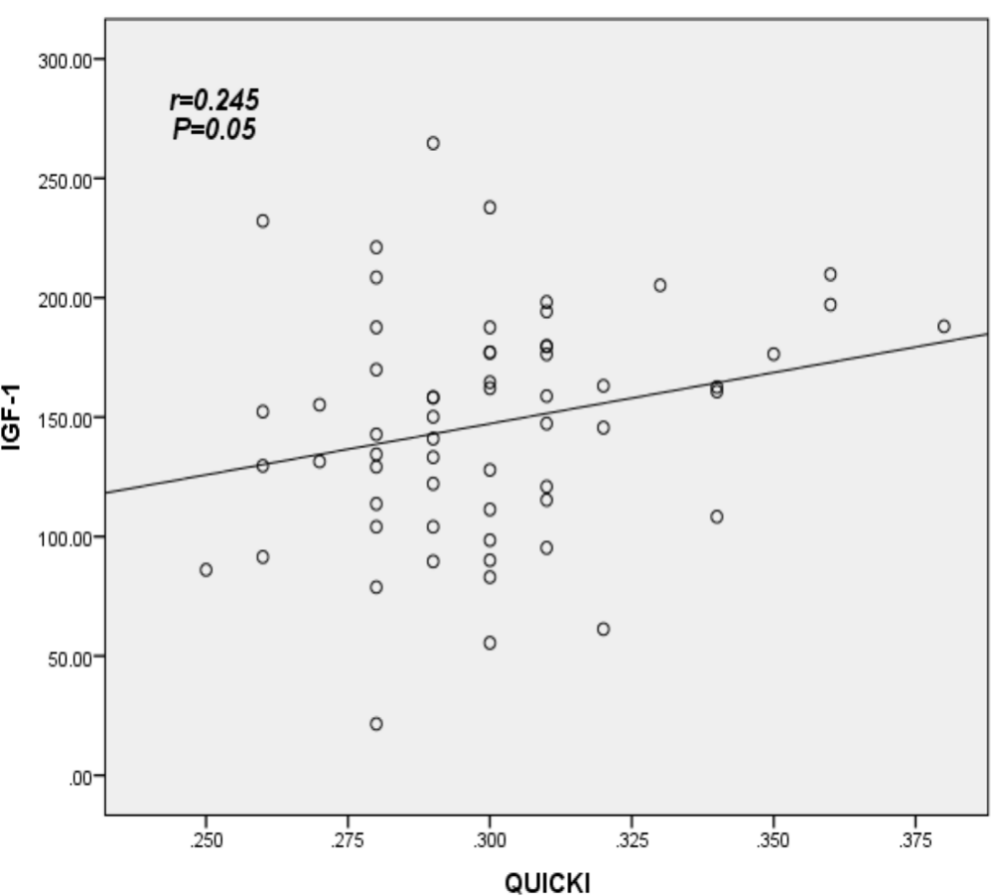
Correlation between IGF-1 and factor related diabetes QUICKI; using Spearman’s rank correlation coefficient; *P significant at level ≤ 0.05

The primary demographic data are presented in Table 1. The mean Hemoglobin A1c (HbA1c) for the sample is 8.6%, with an average diabetes duration of 6.6 years and a mean participant age of 53.38 years. Elevated levels of Insulin-Like Growth Factor-1 (IGF-1) were found to be associated with increasing age, longer duration of Diabetes Mellitus (DM), higher HbA1c, and higher Quantitative Insulin Sensitivity Check Index (QUICKI) value (*P* = 0.050, *P* = 0.044, *P* = 0.001, and *P* = 0.039 respectively). Additionally, higher IGF-1 levels were associated with a lower Body Mass Index (BMI) (*P* = 0.004). However, IGF-1 levels did not show a significant association with other parameters, including fasting Growth Hormone (GH), Homeostatic Model Assessment for Insulin Resistance (HOMA-IR), Homeostatic Model Assessment for β-cell function (HOMA-B), Waist-Hip Ratio (WHR), fasting Plasma Glucose (PG), fasting Triglycerides (TG), and fasting insulin, as depicted in Table 2.As depicted in Table 3, poorly controlled diabetes, characterized by Hemoglobin A1c (HbA1c) levels exceeding 8, was significantly associated with elevated levels of Insulin-Like Growth Factor-1 (IGF-1) compared to diabetes that was fairly controlled (HbA1c < 8). The data also revealed that patients with a normal Homeostatic Model Assessment for Insulin Resistance (HOMA-IR) exhibited a significantly higher mean IGF-1 compared to those with a high HOMA-IR (> 2.5), with values of 192.83 versus 143.85, respectively (*P* = 0.001). However, no significant difference was observed in the mean IGF-1 when using the Quantitative Insulin Sensitivity Check Index (QUICKI). IGF-1 demonstrated a significant negative correlation with Body Mass Index (BMI) (*r*= -0.377, *P* = 0.003), and a significant positive correlation with HbA1c and QUICKI (*r* = 0.312, *P* = 0.015 and *r* = 0.245, *P* = 0.05 respectively). These relationships are further illustrated in Table 4; Figs. 1, 2 and 3.

**Table 1:** Mean and standard deviation of studied sample according to their characteristic features and biological tests

*N*	60
Age (year)	53.3 ± 9.6
Duration of T2DM (year)	6.6 ± 5
Body Mass Index (kg/m²)	34 ± 5.1
Waist/Hip Ratio	0.98 ± 0.07
Fasting Plasma Glucose (mg/dl)	142.8 ± 43.2
HbA1c (%)	8.6 ± 1.8
Fasting Triglyceride (mg/dl)	171.2 ± 84.8
Fasting Growth Hormone (ng/ml)	0.5 ± 1
IGF-1 (ng/ml)	147.1 ± 48
Fasting insulin (mIU/ml)	20.2 ± 13.7
HOMA-IR	6.5 ± 4.2
HOMA-B	154.1 ± 206.9
QUICKI	0.3 ± 0.02

**Table 2:** Mean distribution of studied sample according to IGF-1 and their characteristic and biological features

Variables	IGF-1	No.	Mean	SD	*P*
Age (Year)	Normal	46	52.13	9.72	**0.050**
	High	14	57.5	8.35	
Duration of T2DM (year)	Normal	46	5.98	4.92	**0.044**
	High	14	8.64	4.91	
BMI (kg/m²)	Normal	46	34.94	5.18	**0.004**
	High	14	31.04	3.73	
WHR	Normal	46	0.98	0.07	0.885
	High	14	0.98	0.07	
FPG (mg/dl)	Normal	46	142.96	44.36	0.95
	High	14	142.2	40.80	
Fasting TG (mg/dl)	Normal	46	164.5	81.8	0.3
	High	14	193.3	94.15	
Fasting GH (ng/ml)	Normal	46	0.52	1.07	0.66
	High	14	0.66	1.03	
HbA1c (%)	Normal	46	8.09	1.32	**0.001**
	High	14	10.15	1.74	
Fasting insulin (mIU/ml)	Normal	46	21.21	14.38	0.25
	High	14	16.87	11.26	
HOMA-IR	Normal	46	6.81	4.24	0.31
	High	14	5.47	4.22	
HOMA-B	Normal	46	157.2	212.7	0.83
	High	14	144.3	193.94	
QUICKI	Normal	46	0.29	0.02	**0.039**
	High	14	0.31	0.03	

*Independent t test, P is significant at level ≤ 0.05

**Table 3:** Mean IGF-1 of studied sample in Association with Diabetes control, insulin resistance/sensitivity and gender

Variables		No.	IGF-1 Mean	SD	P*
HbA1c	< 8	37	134.9	42.54	0.010
	> 8	23	166.8	50.78	
HOMA IR	Normal	4	192.83	14.14	0.001
	IR	56	143.85	48.004	
QUICKI	0.30–0.45	33	152	45.21	0.39
	< 0.30	27	141.2	51.57	
Gender	Male	40	146.12	47.27	0.822
	Female	20	149.12	50.79	

*Independent t test, P is significant at level ≤ 0.05

**Table 4:** Correlation between different features and biological tests of studied sample (Age Group, Duration DM, BMI, WHR, FPS, HbA1c, TG, GH, F. insulin, IGF-1, HOMA-IR, HOMA-B, and QUICKI)

		Duration	BMI	WHR	FPG	HbA1c	TG	GH	IGF-1	INSULIN	HOMA-IR	HOMA-B	QUICKI
**Age/ Year**	**r**	0.258*	− 0.130-	0.035	0.187	0.014	− 0.068-	0.368**	− 0.139-	− 0.174-	− 0.119-	− 0.006-	0.051
	**P**	0.046	0.321	0.79	0.152	0.915	0.604	0.004	0.291	0.185	0.363	0.964	0.699
**Duration**	**r**	1	− 0.294-*	0.093	0.427**	0.479**	0.166	0.057	0.186	− 0.286-*	0.026	− 0.124-	0.001
	**P**		0.023	0.48	0.001	0.000	0.204	0.668	0.155	0.027	0.845	0.346	0.996
**BMI**	**r**		1	− 0.088-	− 0.102-	0.053	− 0.079-	0.027	− 0.377-**	0.380**	0.303*	− 0.004-	− 0.285-*
	**P**			0.506	0.436	0.689	0.547	0.838	*0.003*	0.003	0.019	0.977	0.027
**WHR**	**r**			1	0.304*	0.415**	0.062	− 0.350-**	− 0.003-	0.02	0.217	0.079	− 0.265-*
	**P**				0.018	0.001	0.639	0.006	0.983	0.88	0.096	0.547	0.041
**FPG**	**r**				1	0.682**	0.509**	− 0.024-	− 0.064-	− 0.133-	0.326*	− 0.339-**	− 0.386-**
	**P**					0.000	0.000	0.853	0.626	0.312	0.011	0.008	0.002
**HbA1c**	**r**					1.000	0.290	− 0.014-	*0.312*	− 0.031-	0.326*	− 0.123-	− 0.386-*
	**P**						0.025	0.917	*0.015*	0.814	0.036	0.351	0.041
**TG**	**r**						1	− 0.231-	0.071	0.093	0.287*	− 0.369-**	− 0.281-*
	**P**							0.076	0.589	0.479	0.026	0.004	0.03
**GH**	**r**							1	0.051	− 0.349-**	− 0.453-**	− 0.012-	0.381**
	**P**								0.699	0.006	0.000	0.927	0.003
**IGF-1**	**r**								1	− 0.128-	*− 0.211-*	− 0.013-	*0.245*
	**P**									0.329	*0.106*	0.923	*0.05*
**INSULIN**	**r**									1	0.798**	0.281*	− 0.799-**
	**P**										0.001	0.029	0.001
**HOMA-IR**	**r**										1	0.054	− 0.943-**
	**P**											0.681	0.001
**HOMA-B**	**r**											1	− 0.071-
	**P**												0.592
. .													

*Correlation is significant at level ≤ 0.05

**Correlation is significant at level ≤ 0.01

## Discussion

This study assessed the serum concentrations of total Insulin-Like Growth Factor-1 (IGF-1) in patients with Type 2 Diabetes Mellitus (T2DM) and examined its relationship with insulin resistance. The findings revealed a significant reduction in serum IGF-1 levels in patients with an escalating Body Mass Index (BMI). Furthermore, higher serum IGF-1 levels were observed in patients with poorly controlled T2DM, characterized by Hemoglobin A1c (HbA1c) levels exceeding 8%. Notably, T2DM patients with a high Insulin Resistance (IR) index, as measured by the Homeostatic Model Assessment for Insulin Resistance (HOMA-IR), exhibited lower IGF-1 levels compared to those with normal IR. These results underscore the complex interplay between IGF-1, BMI, glycemic control, and insulin resistance in T2DM.Our study revealed a significant inverse correlation between Insulin-Like Growth Factor-1 (IGF-1) levels and Body Mass Index (BMI) in patients with diabetes (*P* = 0.003). This observation aligns with prior research, which has reported a marked reduction in IGF-1 levels in obese patients with Type 2 Diabetes Mellitus, in comparison to obese non-diabetic patients and lean control subjects. This underscores the complex interplay between obesity, diabetes, and IGF-1 levels. [14, 37, 38]. Aleidi et al. conducted a study on Insulin-Like Growth Factor-1 (IGF-1) in obese patients with diabetes, demonstrating significantly lower IGF-1 levels compared to obese non-diabetic individuals [13]. It is well-established that obesity is associated with a decrease in Growth Hormone (GH) secretion, a condition that is reversible with weight loss [27, 39]. Despite the reduced GH levels in obesity, studies have reported inconsistent data concerning the levels of circulating total IGF-1. For instance, total IGF-1 levels have been reported as either low or not significantly different in obese subjects compared to those of normal weight [40–43]. Overall, the simultaneous effects of diabetes and obesity on IGF-1 levels are complex, as changes in the GH/IGF-1 axis induced by obesity oppose those triggered by metabolic processes in Type 2 Diabetes Mellitus (T2DM) [20]. A study conducted by Bushra F. Hasan et al. found that IGF-1 levels were significantly lower in obese T2DM patients compared to non-obese T2DM patients. This finding aligns with our results, which demonstrated lower IGF-1 levels in patients with increased Body Mass Index (BMI) [15].Our investigation revealed a significant and positive correlation between Insulin-Like Growth Factor-1 (IGF-1) levels and glycated haemoglobin (HbA1c) (*P* = 0.015). Notably, IGF-1 concentrations were found to be elevated in patients with uncontrolled diabetes mellitus, characterized by HbA1c levels exceeding 8% [44]. This observation aligns with the findings of El-Mesallamy HO et al., thereby corroborating our results [14]. Intriguingly, IGF-1 levels were significantly higher in patients with uncontrolled diabetes. This phenomenon can largely be attributed to the insulin-like effect of IGF-I in maintaining euglycemia in the face of escalating insulin resistance (IR). As IR progresses and glycemic control deteriorates, a compensatory up-regulation of hepatic IGF-I production ensues, leading to a subsequent increase in free (bioactive) IGF-I levels [19].

Our study, employing multivariate linear regression, revealed a statistically significant correlation between Insulin-Like Growth Factor-1 (IGF-1) levels and the Quantitative Insulin Sensitivity Check Index (QUICKI) (*P* = 0.05). In contrast, Suda K et al. reported no correlation between QUICKI and IGF-1 levels [45]. This discrepancy may be attributed to the influence of various medications used in the treatment of diabetic patients on QUICKI. Our findings also align with the observation of lower mean IGF-1 levels in individuals with high Homeostatic Model Assessment for Insulin Resistance (HOMA-IR), suggesting a role for IGF-1 in insulin resistance in patients with Type 2 Diabetes Mellitus. IGF-1 has been successfully employed in the treatment of certain rare lipodystrophic forms of Type 2 Diabetes in individuals with severe insulin resistance. However, these findings should be interpreted with caution, as total IGF-1 may not be the optimal indicator for tracking the relationship between glycemic control and insulin resistance. Subcomponents of IGF-1 have been demonstrated to play distinct roles in glucose metabolism. For instance, S.N Rajpathak et al. suggest that low levels of Insulin-Like Growth Factor Binding Protein-1 (IGFBP-1) and possibly IGFBP-2, along with high free IGF-1, may all exert an adaptive effect on insulin resistance. Conversely, IGFBP-3 might represent a factor associated with a higher risk of insulin resistance and Type 2 Diabetes [40].

### Limitation points

The study employed a cross-sectional design as opposed to a prospective one, and the sample size recruited for the study was relatively small. Additionally, it is important to note that the Quantitative Insulin Sensitivity Check Index (QUICKI) and the Homeostatic Model Assessment (HOMA) models may be influenced by the medications used by the participants. Moreover, Growth Hormone (GH) measurements were conducted in a random state rather than under stimulated conditions. These factors should be taken into consideration when interpreting the results of the study.

## Conclusions and recommendations

Growth Hormone (GH) and Insulin-Like Growth Factor-1 (IGF-1) play intricate roles in the pathophysiology of Type 2 Diabetes Mellitus (T2DM). The present study observed that IGF-1 levels were diminished in obese patients, while they were elevated in cases of uncontrolled diabetes. Furthermore, an increase in insulin resistance was associated with a decrease in IGF-1 levels. These findings suggest that targeting IGF-1 in T2DM could be explored as a potential therapeutic strategy in the foreseeable future. However, this proposition necessitates further investigation through meticulously designed prospective randomized controlled trials.

## Data Availability

We hereby declare that all data are available upon request from Araz Al-Saffar who can be contacted on arazbassim@gmail.com.

## Abbreviations

GH: Growth Hormone
IGF-1: Insulin-Like Growth Factor one
T2DM: Type 2 Diabetes Mellitus
HOMA-IR: Homeostasis Model Assessment of Insulin Resistance
HOMA-B: Homeostasis Model Assessment of Beta cell function
QUICKI: Quantitative Insulin Sensitivity Check Index
FPG: Fasting Plasma Glucose
T1DM: Type 1 Diabetes Mellitus
HbA1c: Glycated haemoglobin
BMI: Body Mass Index
WHR: Waist Hip Ratio
TG: Triglyceride
GHRH: Growth Hormone Releasing Hormone
IGFBP-1: Insulin Like Growth Factor Binding Protein-1
ECLIA: ElectroChemiLuminescence ImmunoAssay
IR: Insulin Resistance
DM: Diabetes Mellitus
HPLC: High Performance Liquid Chromatography
